# Flow Field Simulation and Experimental Study of Electrode-Assisted Oscillating Electrical Discharge Machining in the C_f_-ZrB_2_-SiC Micro-Blind Hole

**DOI:** 10.3390/ma18173944

**Published:** 2025-08-22

**Authors:** Chuanyang Ge, Sirui Gong, Junbo He, Kewen Wang, Jiahao Xiu, Zhenlong Wang

**Affiliations:** 1 Dalian Scientific Test and Control Technology Institute, Dalian 116013, China; 565237147@163.com (J.H.); heaven996@hotmail.com (K.W.); xiujiahao163@163.com (J.X.); 2 Beijing Aeronautical Technology Research Center, Beijing 100076, China; 19b908132@stu.hit.edu.cn; 3 School of Mechatronics Engineering, Harbin Institute of Technology, Harbin 150001, China

**Keywords:** C_f_-ZrB_2_-SiC, micro-EDM, micro-hole, flow field simulation, electrode-assisted oscillating device

## Abstract

In the micro-EDM blind-hole machining of C_f_-ZrB_2_-SiC ceramics, defects such as bottom surface protrusion and machining fillets are often encountered. The implementation of an electrode-assisted oscillating device has proven effective in improving machining outcomes. To unravel the fundamental reasons behind the optimization enabled by this auxiliary oscillating device, this paper presents fluid simulation research, providing a quantitative comparison of the differences in machining gap flow field characteristics and debris motion behaviors under conditions with and without the assistance of the oscillating device. Firstly, this paper briefly describes the characteristics of C_f_-ZrB_2_-SiC discharge products and flow field deficiencies during conventional machining and introduces the working principle of electrode-assisted oscillation devices to establish the background and objectives of the simulation study. Subsequently, this research established simulation models for both conventional machining and oscillating machining based on actual processing conditions. CFD numerical simulations were conducted to compare flow field differences between conditions with and without auxiliary machining devices. The results demonstrate that, compared to conventional machining, electrode oscillation not only increases the maximum velocity of the working fluid by nearly 32% but also provides a larger debris accommodation space, effectively preventing secondary discharge. Regarding debris agglomeration, oscillating machining resolves the low-velocity zone issues present in conventional modes, increasing debris velocity from 0 mm/s to 7.5 mm/s and ensuring continuous debris motion. Furthermore, the DPM was used to analyze particle distribution and motion velocities, confirming that vortex effects form within the hole under oscillating conditions. These vortices effectively draw bottom debris outward, preventing local accumulation. Finally, from the perspective of debris distribution, the formation mechanisms of micro-hole morphology and the tool electrode wear patterns were explained.

## 1. Introduction

As a novel ultra-high-temperature ceramic composite material, C_f_-ZrB_2_-SiC is reinforced with carbon fibers and SiC particles, with ZrB_2_ serving as the matrix [1]. The combination of these three phases endows the material with high specific strength, high toughness, superior thermal shock resistance, and outstanding high-temperature oxidation resistance [2,3]. Consequently, this composite has emerged as an ideal candidate for next-generation aerospace thermal components. Its excellent performance demonstrates broad application potential in high-temperature environments, including aircraft leading edges, nose cones, rocket engine systems, and micro-thruster hot-end components [4,5,6], garnering significant attention in both fundamental research and engineering domains.

With advancing technologies, the miniaturization trends in MEMS and microfluidic systems impose increasingly stringent requirements on the microstructural processing of composite materials [7,8]. For instance, precision machining of micro-hole structures in C_f_-ZrB_2_-SiC composites faces multiple high-demand application scenarios requiring extreme accuracy and quality. However, the material’s extreme hardness and the low fracture toughness of its ceramic phases create secondary processing challenges in conventional machining processes, including severe tool wear, low machining efficiency, and difficulties in maintaining surface quality integrity [9,10].

Micro-electrical discharge machining (micro-EDM) is a non-contact, specialized machining method that leverages the localized transient high temperatures generated by discharges between the tool electrode and the workpiece to remove material through non-mechanical forces; thereby, this technology is not limited by material hardness and strength [11,12]. Concurrently, given the low fracture toughness of the ceramic phase in such materials, EDM technology is more suitable for processing such materials due to its characteristic of not generating macroscopic forces. According to the author’s prior research, micro-EDM has been experimentally validated as an effective means for machining such material [13].

However, in the field of micro-hole machining, it is difficult to avoid defects such as excessive machining taper and tool electrode deformation when using micro-EDM. To solve the processing defects of difficult-to-machine materials or optimize the shape accuracy of specific structures, it is often necessary to combine auxiliary devices to improve processing accuracy and efficiency. Auxiliary devices usually include various auxiliary methods such as electrode motion assistance, workpiece motion assistance, expansion of machining degrees of freedom, electrode updating, and interelectrode state optimization.

For instance, to address electrode renewal and optimize electrode motion, Li et al. [14] implemented combined rotation and feed motions for large-length tool electrodes using a friction-drive micro-feed servo mechanism and an electrode rotary feed mechanism. This approach resolved the issue of electrode wear in micro-hole machining, improved machining efficiency and precision, and enhanced the reliability and stability of electrode wire travel, achieving electrode wire travel accuracy within a 2 μm tolerance. To optimize spindle motion and enable rapid adjustment of the discharge gap, Feng et al. [15,16] developed a high-performance magnetically levitated spindle for EDM characterized by low load inertia and high response frequency, integrated with incomplete differential lead-correction PID control to achieve precise motion regulation. This spindle ultimately achieved a spindle positioning accuracy of ±1 μm, optimizing machining efficiency, electrode wear, and machining taper. To improve the interelectrode debris discharge state during machining, Tong et al. [17] proposed an auxiliary machining method that integrates piezoelectric ceramics with the workpiece fixture, enabling the workpiece to reciprocate along the spindle axis during machining. This method, akin to high-frequency tool lifting motion, ultimately optimized the machining results. Endo et al. [18] studied the application of piezoelectric ceramics between the workpiece and the motion platform to generate continuous axial reciprocal vibration perpendicular to the spindle axis during milling, thereby altering the gap size to promote debris discharge. Zhang et al. [19] applied this vibration to the tool end during micro-hole machining, increasing the EDM efficiency of TC4 material by 2.4 times while significantly improving tool wear and machining taper. From the perspective of machining taper control, although the aforementioned studies improved machining outcomes, they did not directly control the machining taper. In contrast, Tong et al. [20] designed a spindle structure capable of adjusting the machining taper, achieving machining taper control with a taper angle ranging from 0 to 1.3°.

In the author’s prior research, an electrode-assisted oscillating device was developed and experimentally demonstrated to significantly enhance machining efficiency and depth-to-diameter ratio. The working principle of the electrode-assisted oscillating device involves controlling a rod-shaped tool electrode through two guides. The lower guide remains fixed, while the upper guide rotates eccentrically, enabling the tool electrode to perform a conical oscillating motion. The electrode’s oscillation angle can be adjusted by modifying the eccentricity and spacing of the two guides. Experimental validation has demonstrated that, compared to conventional rotating electrodes, this motion mode significantly enhances debris evacuation, thereby exhibiting superior machining efficiency and quality. Furthermore, in contrast to electrode vibration machining, this approach allows for the compensation and adjustment of machining taper control. However, previous studies have only experimentally validated the effectiveness of this machining method, without clearly revealing the fundamental reasons behind such optimized outcomes. The most crucial issue is that the actual interelectrode gap flow field conditions remain indistinct and difficult to observe. In a word, the underlying mechanisms driving these improvements remain to be elucidated [21].

This paper addresses the machining challenges of C_f_-ZrB_2_-SiC blind holes by analyzing the flow field characteristics of an electrode-assisted oscillating mechanism. Through comparative studies of flow field states and debris distribution with and without auxiliary oscillation, the essential reason underlying machining optimization is revealed. In the beginning, this paper briefly describes the characteristics of C_f_-ZrB_2_-SiC discharge products and flow field deficiencies during conventional machining and introduces the working principle of electrode-assisted oscillation devices to establish the background and objectives of the simulation study. Computational fluid dynamics (CFD) simulations of the oscillating machining gap flow field are conducted, with comparative analysis of flow velocity profiles and discrete phase model (DPM) particle distributions. Finally, from the perspective of debris distribution, the formation mechanisms of micro-hole morphology and tool electrode wear patterns are explained.

## 2. Material and Setup

Before conducting fluid simulation research, it is imperative to provide a concise overview of the simulation objectives and the relevant background context. Therefore, this section will first offer a brief description of the equipment and materials utilized in EDM. Based on the machining results of previous studies, this paper elaborates on the C_f_-ZrB_2_-SiC processing problems that occur under traditional processing methods and explains the main difficulties that affect the machining outcomes. Subsequently, this study will introduce the oscillating machining method to be simulated, accompanied by a detailed explanation of the corresponding apparatus. With a clear understanding of the differences in machining processes, a systematic simulation study will then be carried out.

### 2.1. Machining Tool

**Figure 1:**
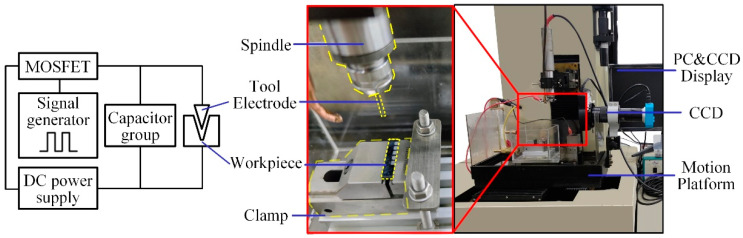
Machine tool structure and power supply schematic diagram.

The micro-EDM machine tool used in the experiment is independently developed by Harbin Institute of Technology. This machine tool system comprises a high-resolution motion stage, a high-speed spindle, a transistor-RC micro-energy pulse power supply, a discharge state detection device, and a servo control system. The modular structure of the machine tool and the schematic diagram of the power supply are shown in Figure 1.

### 2.2. Experimental Materials and Results of Blind-Hole Machining

**Figure 2:**
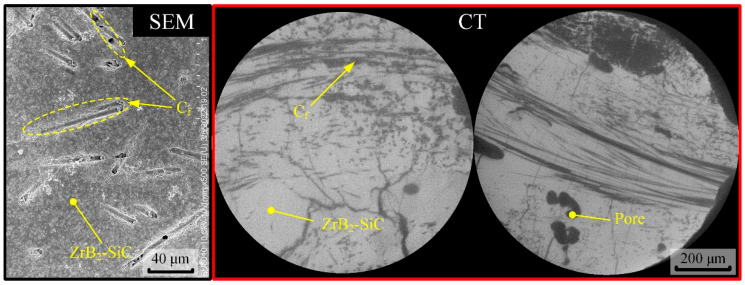
Surface and internal morphology of C_f_-ZrB_2_-SiC ceramic.

As a representative of C_f_-UHTC (carbon-fiber-reinforced ultra-high temperature ceramic) materials, C_f_-ZrB_2_-SiC has excellent mechanical properties. To visually illustrate the morphology and microstructure of the material, the surface and interior of the material were characterized, as shown in in Figure 2, using scanning electron microscopy (FESEM SU8010, HITACHI, Tokyo, Japan) and 3D-CT microscopy (Xradia 520 Versa, Carl Zeiss, Oberkochen, Germany).

**Figure 3:**
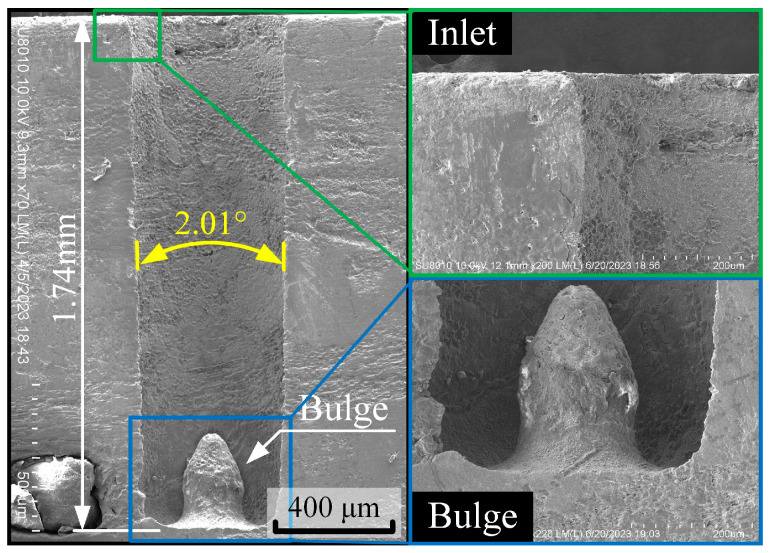
Machining results of C_f_-ZrB_2_-SiC blind hole using micro-EDM.

When the workpiece is chosen as the negative electrode of the power supply and machined in a kerosene dielectric medium through EDM, the blind-hole machining results of the C_f_-ZrB_2_-SiC workpiece can be determined as illustrated in Figure 3. The blind-hole processing result exhibits obvious machining fillets at the entrance and bottom, poor straightness of the hole wall, and significant overcutting. Most importantly, a convex-platform-shaped defect appears at the bottom of the hole [21].

### 2.3. Analysis of Machining Defect Causes

**Figure 4:**
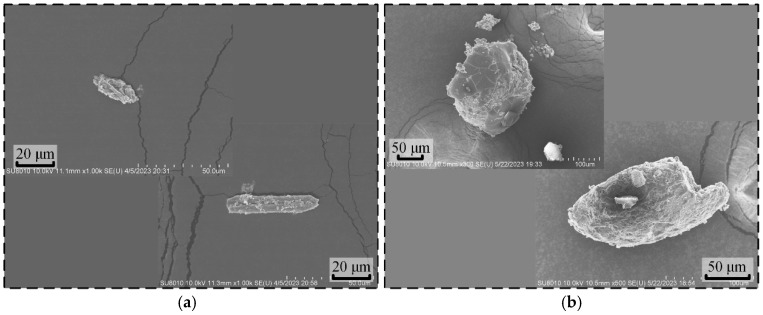
Electro-erosion product of C_f_-ZrB_2_-SiC ceramic. (**a**) Carbon fiber debris. (**b**) Ceramic debris.

When the tool electrode exhibits no significant deformation, workpiece overcutting and bottom surface defects are highly likely to be attributable to issues in the interelectrode flow field. The state of the interelectrode flow field is primarily influenced by the electrical erosion products in the kerosene medium. Therefore, debris generated during micro-hole machining was collected for analysis. Observations revealed a substantial presence of carbon fiber and ceramic fragments within the erosion products, as illustrated in Figure 4.

**Figure 5:**
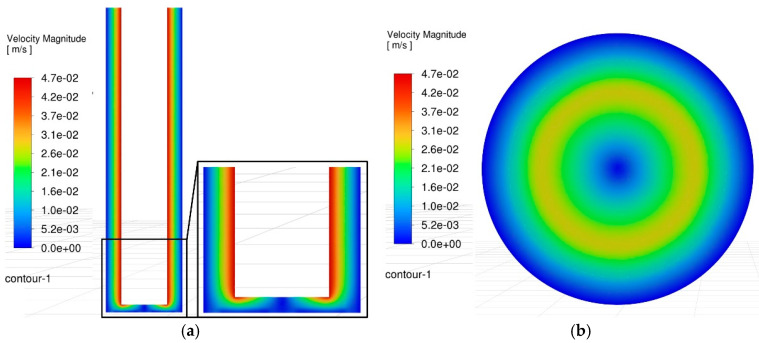
Velocity contour maps in the side and bottom gap flow field. (**a**) Velocity field in the side gap. (**b**) Velocity field in the bottom gap.

To directly analyze the root cause of bottom surface defects, the interelectrode flow field during blind-hole machining was simulated using CFD software (Fluent of Ansys 2024), as shown in Figure 5. The results reveal that, although the rod-shaped electrode provides strong agitation, a distinct low-velocity zone persists near the central region of the hole bottom. This inevitably leads to the accumulation and agglomeration of debris (observed in Figure 4), while simultaneously deteriorating the interelectrode discharge conditions. Consequently, arcing occurs in the bottom region, resulting in debris sintering and the formation of surface protrusions.

### 2.4. Electrode-Assisted Oscillating Device

**Figure 6:**
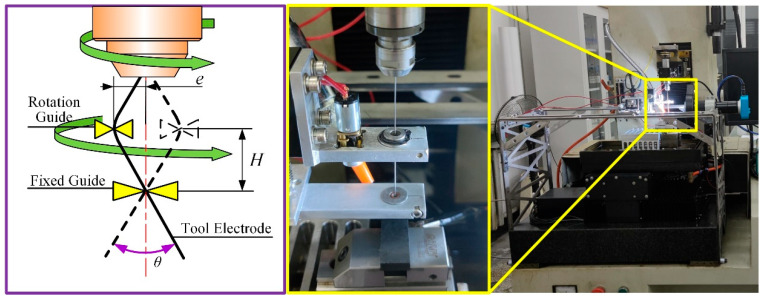
Principle and installation of electrode-assisted oscillating device.

To improve the interelectrode flow field conditions, the authors previously developed a novel auxiliary machining device. This system utilizes dual guide mechanisms to precisely control electrode motion, significantly enhancing agitation effects and thereby improving machining efficiency and quality. The working principle and installation setup are illustrated in Figure 6. The device operates through the coordinated action of a rotary guide and a fixed guide, enabling the rod-shaped electrode to perform conical motion around the spindle’s central axis. By changing the distance (*H*) between these two guides, the oscillating angle of the electrode can be changed. This innovative design fundamentally alters both the kinematic behavior of the tool electrode and its agitation effectiveness in the working area.

## 3. Flow Field Analysis in EDM with an Electrode-Assisted Oscillating Device

When using an electrode-assisted oscillating device for machining, both the debris evacuation space and flow field behavior differ fundamentally from those in conventional rod-shaped electrode machining. To examine the flow field disparities between processes with and without the auxiliary oscillating device, this study conducts CFD simulations of the gap flow field under oscillation machining and contrasts it with conventional methods. These differences are visualized through flow field velocity profiles and DPM particle distribution patterns.

To conduct CFD simulations, it is necessary to establish an appropriate numerical model and select suitable computational methods. Specifically, the flow field simulation process based on simulation software can be divided into three main stages: pre-processing, solver setup, and post-processing. The tasks involved in the pre-processing stage include establishing the computational domain for simulation, generating a mesh for the computational domain, defining the physical properties of the fluid, and determining the computational boundary and initial conditions. Building upon the pre-processing stage, the solver defines the boundary conditions, physical properties, computational methods, and initial conditions and then solves the governing equations. Post-processing involves outputting the results of the simulation calculations. In this study, the primary focus is on the axial and radial cross-sections of the flow field within the hole. We explore information such as the velocity distribution and particle distribution in these two cross-sections and output velocity contour plots, XY scatter plots, and particle distribution plots. When establishing the CFD model, to simplify the modeling and computational processes, the model is developed based on the following assumptions: firstly, in the actual machining process, electrode wear occurs over time, but for the sake of simplifying the model and focusing on the fluid-flow characteristics, the electrode shape in the simulation is set as an ideal shape. Secondly, due to the very small force generated by discharge during the EDM process, the influence of discharge on electrode movement is not considered. Thirdly, because the pressure changes in the machining gap are relatively small, and the density of kerosene can be considered constant under these conditions, the fluid is incompressible. Fourthly, the morphology and generation process of debris is simplified, treating it as regular particles of uniform size and generating them from the bottom surface.

### 3.1. Model Establishment and Computational Methods

#### 3.1.1. Initial Model Establishment

**Figure 7:**
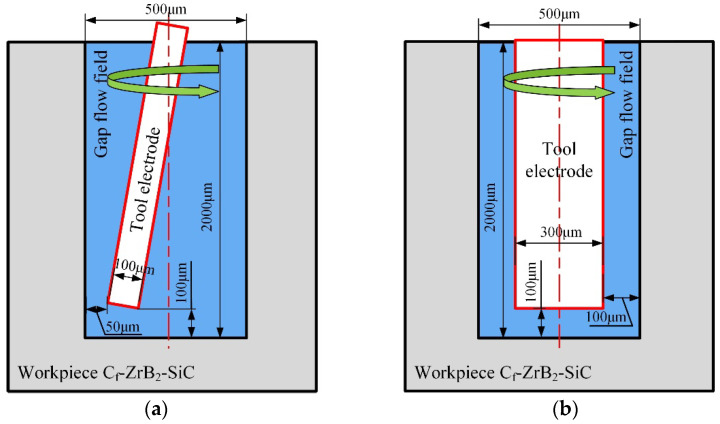
Schematic diagram of flow field simulation model for oscillating machining and conventional machining. (**a**) Schematic diagram of oscillating machining flow field simulation model. (**b**) Schematic diagram of conventional machining flow field simulation model.

The target hole diameter is set to 0.5 mm, and the simulation focuses on the gap flow field at a depth of 2 mm. Schematic illustrations of the flow field models for both scenarios are provided in Figure 7.

For the control group’s flow field model, a tungsten rod electrode with a diameter of 300 μm was set. Debris analysis indicated that the relatively large size of machining debris resulted in an enlarged side machining gap. Consequently, the side gap was set to 100 μm in the flow field simulation. As the bottom surface served as the primary discharge region with more intensive debris generation, its gap was set to 100 μm. An oscillation-assisted machining flow field model was then established to simulate the flow conditions during the EDM of the 500 μm micro-hole using a 100 μm tungsten electrode. The oscillating motion facilitated better debris evacuation, leading to a smaller discharge gap compared to conventional machining. Thus, the side gap was set to 50 μm, with an oscillation angle (*θ*) of about 9°. The bottom gap remained at 100 μm, as it continued to be the dominant material removal zone. Based on the aforementioned dimensions for the hole wall and tool electrode, the model could be divided into two parts: the static domain and the dynamic domain, with proper boundary demarcation.

#### 3.1.2. Meshing

During the meshing process, to ensure the accuracy and reliability of the simulation results, adjustments were made to the global mesh settings. A combination of proximity sizing control and curvature control was employed to optimize the mesh size. Specifically, both the minimum size for capturing curvature and the minimum size for proximity were set to approximately 2.67 μm, enabling effective capture of variations within the flow field. Inflation layers were added to the electrode surface, with a smooth transition selected as the inflation type to avoid numerical errors caused by abrupt mesh changes.

The static domains of both the oscillating machining model and the control model have regular shapes. Scanning meshes were generated for these domains, with triangular mesh types chosen to ensure uniform mesh quality for regular-shaped static domains.

#### 3.1.3. Boundary Definition

The tool electrode, as a moving component during the machining process, has its boundary condition settings reflecting the actual motion state and fluid interaction characteristics. In the wall settings, due to the rotational motion of the tool electrode, it was designated as a “Moving Wall” with a rotational motion type. The relative motion was described as “Relative to Adjacent cell zone.” Additionally, assuming no relative sliding between the fluid and the tool electrode surface, the shear condition was set to “No slip” to simulate the characteristic of fluid adhering to the solid surface. Considering that the tool electrode surface is not absolutely smooth and has a certain roughness, the wall roughness was set to 0.5.

For the hole wall of the micro-hole, due to the fixed wall surface, it was set as “Stationary Wall.” In terms of shear conditions and wall roughness, it is similar to the tool electrode.

Furthermore, during the EDM process, the temperature of the working fluid generally does not undergo significant changes, and the influence of instantaneous thermal effects between the electrodes on the flow field is typically not considered in flow field simulations. Therefore, with regard to thermal boundary conditions, issues related to heat exchange or temperature variations were not taken into account.

#### 3.1.4. Physical Model and Simulation Solver Settings

During the solving process, the standard k-ε model was employed to simulate the complex flow conditions within the machining gap. The specific solving strategy was as follows: First, a steady-state calculation mode was utilized. When the steady-state calculation converged and a relatively stable flow field was obtained, transient calculations were conducted to capture the dynamic characteristics of the flow field. Subsequently, the DPM was incorporated to simulate the debris particles generated during the machining process. It is capable of considering the forces acting on the debris particles in the flow field as well as the interactions between particles and the fluid and between particles and the wall. This enables accurate simulation of the motion and distribution of debris particles within the machining gap.

The standard k-ε model was selected as the turbulence model. This process mainly involves two key equations for turbulent kinetic energy (k) and turbulent dissipation rate (ε). For incompressible media, the k equation is expressed as follows:

(1) $$\rho \frac{\partial k}{\partial t}+\rho \frac{\partial (ku_{j})}{\partial x_{j}}=\frac{\partial }{\partial x_{j}}\left[\left(\mu +\frac{\mu_{t}}{\sigma_{k}}\right)\frac{\partial k}{\partial x_{j}}\right]+2\mu_{t}S_{ij}\cdot S_{ij}-\rho \varepsilon$$

Here, *ρ* represents the density of the working fluid, *u* is the fluid velocity, *x* denotes the spatial coordinate, *i* and *j* indicate the directional component, *μ* is the dynamic viscosity of the fluid, and *μ_t_* is the turbulent viscosity, which takes a value of 0.09 in the standard k-ε model. *σ*_k_ is the Prandtl number for the turbulent kinetic energy k, *S_ij_* is the strain rate tensor, and *ε* is the turbulent dissipation rate.

The turbulent dissipation rate ε equation simplifies for incompressible media as follows:

(2) $$\rho \frac{\partial \varepsilon }{\partial t}+\rho \frac{\partial (\varepsilon u_{j})}{\partial x_{j}}=\frac{\partial }{\partial x_{j}}\left[\left(\mu +\frac{\mu_{t}}{\sigma_{\varepsilon }}\right)\frac{\partial \varepsilon }{\partial x_{j}}\right]+2{C_{1\varepsilon }\frac{\varepsilon }{k}\mu }_{t}S_{ij}\cdot S_{ij}-C_{2\varepsilon }\rho \frac{\varepsilon^{2}}{k}$$

Here, *σ*_ε_ is the Prandtl number for the turbulent dissipation rate ε, typically taking a value of 1.3. *C*_1*ε*_ and *C*_2*ε*_ are model constants, usually set to 1.44 and 1.92, respectively.

By solving the aforementioned k and ε equations, in conjunction with the continuity equation (Equation (3)) and momentum equation (Equation (4)) for incompressible fluids, the distributions of physical quantities such as velocity, pressure, turbulent kinetic energy, and turbulent dissipation rate in the flow field of an incompressible medium can be obtained. This provides flow field information for subsequent DPM simulations, enabling the simulation of fluid flow and debris particle motion within the gap.

(3) $$\frac{\partial u_{i}}{\partial x_{i}}=0$$

(4) $$\rho \frac{\partial u_{i}}{\partial t}+\rho \frac{\partial (u_{i}u_{j})}{\partial x_{j}}=-\frac{\partial p}{\partial x_{i}}+\frac{\partial }{\partial x_{j}}\left[\left(\mu +\mu_{t}\right)(\frac{\partial u_{i}}{\partial x_{j}}+\frac{\partial u_{j}}{\partial x_{i}})\right]$$

In the parameter settings of the discrete phase model (DPM), since the density and geometric shape of the debris remain constant throughout the simulation, they are simplified as inert particles with a characteristic diameter of 2.5 μm. The initial injection location (inlet) of these particles is positioned at the bottom face of the blind-hole structure. To facilitate subsequent comparative analysis of the debris distribution characteristics in three-dimensional space, an additional initial velocity component is assigned to the particles along the positive Z-axis direction. Regarding the particle tracking strategy, a flow time step-based tracking method is employed to accurately capture the particle motion trajectories. The turbulence parameters at the bottom inlet are specified as follows: a turbulence intensity of 5% and a viscosity ratio of 10. In terms of time step settings, a uniform time step of 1 ms is adopted throughout the simulation.

### 3.2. Simulation Results of the Side Gap Flow Field in Micro-Hole Machining

**Figure 8:**
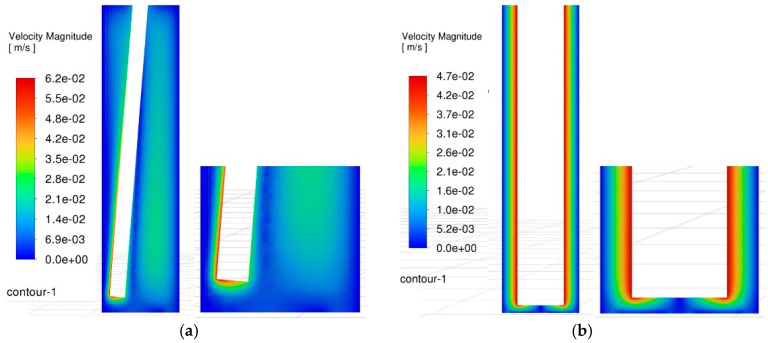
Comparison of velocity contour maps in the side gap flow field. (**a**) Contour plot of velocity field in the side gap during oscillating machining. (**b**) Contour plot of velocity field in the side gap during conventional machining.

Figure 8 compares the velocity contour plots of the side gap flow field under two machining modes, with a spindle speed of 3000 rpm and an oscillating frequency of 60 rpm. In terms of velocity distribution, the gap distribution under conventional machining conditions appears to be more uniform, and the effect of electrode rotation is more pronounced. However, judging from the magnitude of the flow field velocity, it is evident that under oscillating machining conditions, the maximum velocity of the gap flow field can reach 62 mm/s, which is significantly higher than the maximum velocity of 47 mm/s observed in conventional machining. Additionally, under oscillating conditions, the debris containment space is larger, and this area is well agitated, with a relatively high velocity of approximately 26 mm/s, facilitating easier debris ejection. Meanwhile, when the tool electrode undergoes conical motion, it exhibits an equivalent electrode shape that is smaller at the top and larger at the bottom. Therefore, it is inferred that this configuration offers greater advantages in reducing secondary discharges.

### 3.3. Simulation Results of the Bottom Gap Flow Field in Micro-Hole Machining

**Figure 9:**
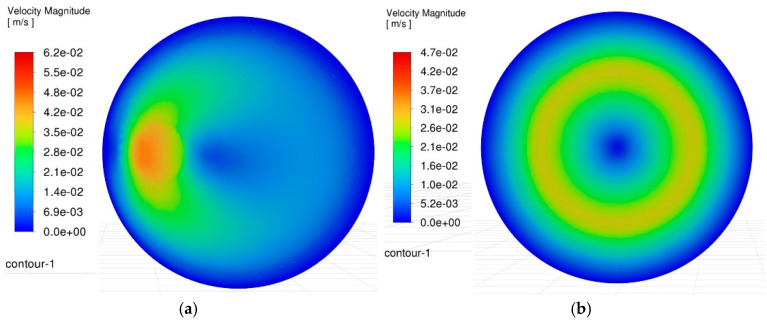
Comparison of velocity contour maps in the bottom gap flow field. (**a**) Contour plot of velocity field in the bottom gap during oscillating machining. (**b**) Contour plot of velocity field in the bottom gap during conventional machining.

The bottom gap of the micro-hole serves as the primary discharge area, and its flow field condition directly impacts the progress of the EDM. Deterioration of the flow field at the bottom gap can lead to issues such as reduced machining efficiency and excessive tool wear. Moreover, it is one of the main reasons for encountering machining limits and defects during micro-hole machining. Therefore, a radial plane was intercepted in the bottom gap flow field region to analyze the differences in the flow field velocity of the two gaps, and the comparison results are shown in Figure 9.

**Figure 10:**
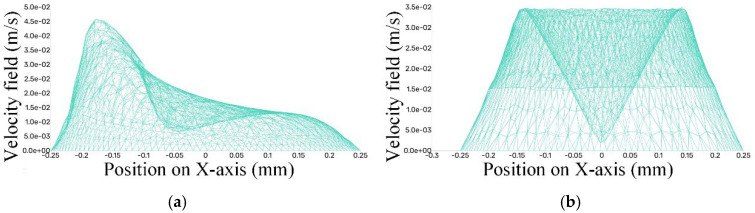
Comparison of velocity data in the bottom gap flow field. (**a**) The data of velocity field in the bottom gap during oscillating machining. (**b**) The data of velocity field in the bottom gap during conventional machining.

Based on the velocity contour plots of the bottom gap flow field, it can be observed that in the conventional machining mode, the flow velocity in the central region of the gap at the bottom of the hole is significantly lower. This phenomenon inevitably leads to the substantial retention and accumulation of machining debris, providing an intuitive explanation for the causes of bottom surface defects in blind-hole machining as mentioned above. In contrast, under the oscillating machining condition, the bottom gap flow field exhibits distinct characteristics. Although there are still low-velocity regions, thanks to the conical oscillating motion of the electrode, the position of this low-velocity area is not fixed but continuously changes around the central axis of the hole. This motion characteristic effectively prevents the local accumulation of debris and plays a crucial role in optimizing the machining results. To more intuitively quantify the velocity field distribution in this plane, the variation curves of the velocity along the X-direction for each node in the flow field were plotted using CFD software, as shown in Figure 10.

The two sets of data clearly demonstrate the differences in flow field velocity between the two machining modes. In the region close to the tool electrode, the area surrounding the oscillating electrode can reach a velocity of 45 mm/s, while the maximum velocity in the area surrounding the electrode under the conventional machining method only reaches 35 mm/s. In the region with the lowest velocity, the flow field velocity under the conventional machining method is nearly zero, but under the oscillating machining method, it still maintains a minimum velocity of 7.5 mm/s.

### 3.4. Analysis of Debris Motion in Micro-EDM Based on the DPM

The motion and distribution pattern of debris play a pivotal role in determining machining efficiency, surface quality, and tool wear in the process. Accurately capturing the dynamic behavior of debris through experimental methods presents significant challenges, as it is difficult to track in real time the motion trajectories of each debris particle and their interactions with the flow field. However, by employing the numerical simulation method based on the DPM within this simulation framework, the distribution characteristics and evacuation mechanisms of electro-erosion products within the machining gap can be effectively revealed solely through simulation. The discrete phase model integrates the Eulerian–Lagrange method. Here, the Eulerian approach is employed to describe the motion of the fluid phase, while the Lagrange method is utilized for tracking the movement of the discrete particle phase. Consequently, this enables quantitative analysis of the velocity, position, and concentration distribution of debris particles, as well as revealing the influence of hydrodynamic effects acting upon them.

To achieve a more accurate simulation of the quantity and volume of debris generated during the EDM process, it is necessary to reasonably define the particle phase parameters, including injection position, injection rate, initial velocity, and drag force effects. Considering the complexity and randomness of debris behavior in actual machining scenarios, this study employs the DPM for simulation while adopting certain simplifications. These simplifications aim to eliminate interference from machining conditions, discharge states, and debris complexity, thereby highlighting critical information such as debris distribution and velocity profiles. The specific simulation scenarios are based on the following assumptions: (1) particle–particle collisions are neglected to conserve computational resources; (2) uniform distribution of electrical erosion products at the hole bottom is assumed under ideal condition of bottom gap discharge; (3) the analysis focuses on ceramic-phase debris, assuming uniform particle size, regular shape, and identical initial velocities generated through electrical erosion removal processes.

When the pulse width and pulse interval are set to 100 μs, ideally, 5000 discharges are generated per second. Based on the single-pulse discharge crater size data presented in Section 2.3, the effective erosion volume per discharge can be determined. Through morphological analysis of electrical erosion products, a mean debris diameter of 2.5 μm is established as the simulation parameter. Therefore, the injection quantity of electro-erosion products can be calculated using Equation (5) based on the particle size and discharge frequency.

(5) $$Q_{\mathrm{Debris}}=\frac{V_{S}\rho }{\frac{4}{3}\pi {\bar{r}}^{3}}\times \frac{1}{T_{on}+T_{off}}$$

In this equation, $Q_{\mathrm{Debris}}$ represents the injection rate of electro-erosion products (kg/s); $V_{S}$ denotes the single-pulse discharge erosion volume (m^3^); $\bar{r}$ is the mean particle diameter (m); *ρ* is the mean debris density of the ceramic phase (kg/m^3^); $T_{on}$ and $T_{off}$ correspond to pulse duration and pulse interval (s).

Furthermore, the initial velocity of electro-erosion debris particles must be specified in the simulation model, with a value of 10 m/s selected for this parameter. When particles move through a fluid, they experience drag force acting opposite to their relative motion with respect to the fluid. Under these conditions, the drag force calculation (Equation (6)) incorporates factors such as fluid density, fluid velocity, particle size, and particle velocity.

(6) $$F_{d}=\frac{1}{8}\pi C_{d}{\bar{d}}^{2}\rho_{l}\left|u_{l}\right.-\left.u_{g}\right|\left(u_{l}-u_{g}\right)$$

Here, $C_{d}$ represents the drag coefficient; $\bar{d}$ denotes the particle diameter (m); $\rho_{l}$ is the fluid density (kg/m^3^); $u_{l}$ and $u_{g}$ correspond to the fluid velocity and particle velocity (m/s), respectively.

**Figure 11:**
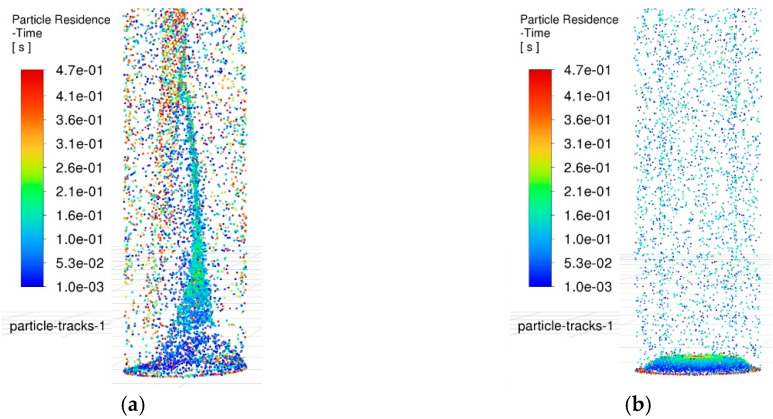
Comparison of particle distribution in the side gap flow field. (**a**) Particle distribution in the side gap flow field during oscillating machining. (**b**) Particle distribution in the side gap flow field during conventional machining.

Based on the aforementioned simulation conditions, transient simulation analysis was performed on the complex flow field described by the discrete phase model, using a time step of 10^−4^ s. After computation and data processing, the simulation results were output as shown in Figure 11. This figure visually illustrates the overall distribution of particles inside the hole when the time step reaches 4700. Additionally, the color gradient and particle density not only represent the spatial positions of the particles within the hole but also reflect the relationship between motion time and their distribution state.

At the time point of 0.47 s, a significant difference is observed in the flow field between the two holes. By analyzing the color gradient of the particles, their residence time within the flow field can be intuitively inferred. Based on the flow field results from oscillating machining, particles eroded at an earlier stage predominantly accumulate in the upper region of the hole, surrounding the tool electrode, and they are likely to be expelled promptly. Conversely, particles eroded at a later stage exhibit vortical distribution patterns due to agitation effects, being dragged from the bottom of the hole to the middle region. Moreover, the particle distribution at the bottom of the hole is uniform, with no obvious agglomeration observed. In contrast, in the control group, the particle distribution markedly differs from that in the oscillating machining group. Newly generated debris agglomerates at the bottom of the flow field. In the middle and upper regions of the hole, the debris density is extremely low, and no newly generated debris has been observed to be drawn into these regions. This finding indicates that oscillating machining effectively promotes debris expulsion through its stirring action and the drag force exerted by vortices.

**Figure 12:**
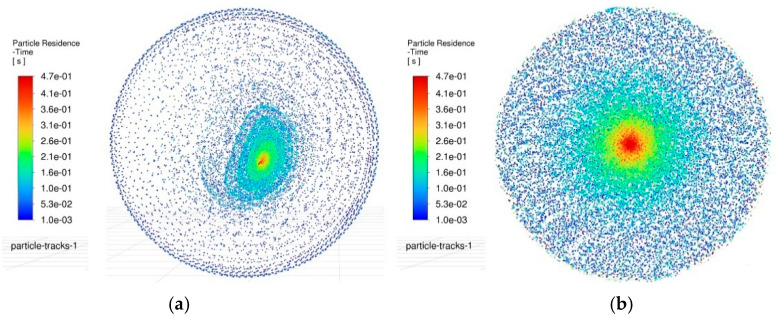
Comparison of particle distribution in the bottom gap flow field. (**a**) Particle distribution in the bottom gap flow field during oscillating machining. (**b**) Particle distribution in the bottom gap flow field during conventional machining.

Furthermore, the retention of debris at the hole bottom also serves as an effective indicator of the machining state. By analyzing the debris distribution in this region, the primary wear zone of the electrode can be inferred, which provides a critical reference for predicting electrode deformation and the morphology of micro-holes. Figure 12 compares the particle distribution in the bottom flow field between the two machining methods at the same time point.

According to the red areas in the diagram, during conventional machining, the debris generated from early electrical discharges remains concentrated and stagnates in the central area of the bottom gap, and a number of particles also linger in the surrounding areas. This undoubtedly hinders the processing progress. In contrast, during oscillating machining, the residual debris on the bottom gap can be reduced, with only slight accumulation observed in a very small area. Moreover, in subsequent machining steps, as the position of the oscillating electrode changes, the debris in these regions disperses accordingly, thereby preventing the prolonged agglomeration of particles.

**Figure 13:**
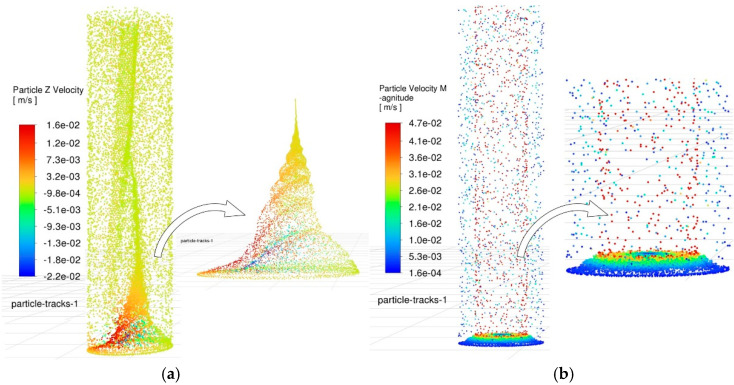
Comparison of particle velocity in the side gap flow field. (**a**) Particle velocity in the side gap flow field during oscillating machining. (**b**) Particle velocity in the side gap flow field during conventional machining.

According to the simulations of debris distribution on the side gap and bottom gap, the debris evacuation dynamics during micro-hole fabrication by EDM can be intuitively understood. This observation corroborates the significant optimization potential of oscillating machining in enhancing process efficiency and quality. Furthermore, the debris velocities induced by electrode oscillation also reflect the potential debris escape capability. As shown in Figure 13, the particle velocities under the current flow field conditions are visualized using a color gradient.

**Figure 14:**
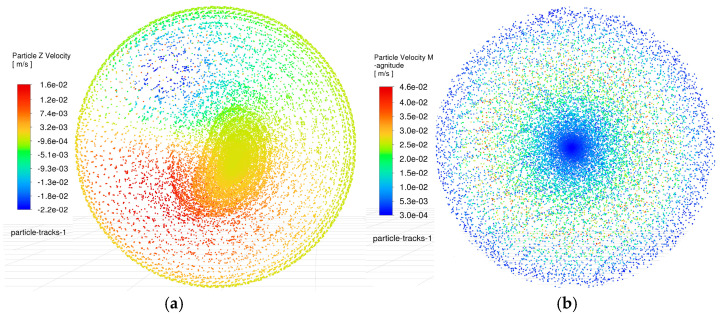
Comparison of particle velocity in the bottom gap flow field. (**a**) Particle velocity in the bottom gap flow field during oscillating machining. (**b**) Particle velocity in the bottom gap flow field during conventional machining.

Color-marked velocity distributions reveal that, during oscillating machining, due to the agitation of the tool electrode, the erosion products at the bottom gap can actually achieve the maximum forward or reverse motion speeds. Thus, the newly generated erosion products can be taken away from the main discharge area rapidly, and it is undoubtedly an ideal situation for debris removal. In contrast to the particle velocity profiles and distribution patterns observed in conventional machining, severe debris retention occurs at both the edge and central regions, which is bound to lead to edge wear of the electrode and central concavity, while also causing noticeable rounded corner and bottom surface protrusion defects in micro-holes. This conclusion is further corroborated by the particle velocity distribution in the bottom gap flow field depicted in Figure 14.

This enables rapid evacuation of freshly generated debris from the primary sparking zone, constituting an ideal debris removal scenario. In contrast, conventional EDM exhibits severe debris retention at both the periphery and center of the machined hole, leading to localized electrode wear (edge rounding) and central recessions, as well as micro-hole defects including fillet rounding and bottom bulging. These observations are further corroborated by bottom surface particle velocity profiles in Figure 14.

### 3.5. Optimization of Micro-Hole Machining for C_f_-ZrB_2_-SiC Based on an Electrode-Assisted Oscillating Device

Simulation results demonstrate that, with an oscillating device, the electrode achieves superior agitation of the gap flow field, effectively preventing debris agglomeration and promoting debris evacuation. Theoretically, this method can effectively enhance processing stability, improve processing efficiency, and expand machining limits.

**Figure 15:**
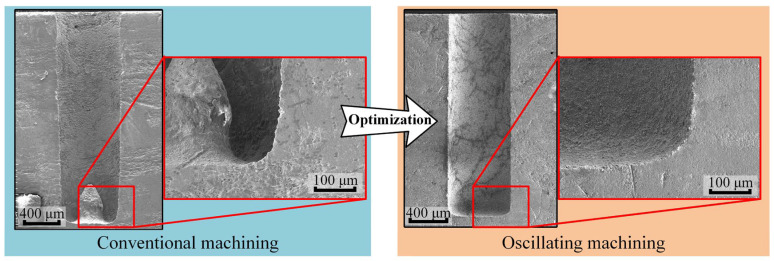
Comparison of blind-hole machining results of oscillating machining and conventional machining.

Based on the experimental findings in the authors’ previous studies, the maximum aspect ratio achieved in conventional machining was approximately 8.4, whereas this value significantly improved to 13.1 when employing an electrode-assisted oscillating device. In the machining quality aspect, defects at the bottom of blind holes were effectively eliminated through this machining method, where no protrusion or severe ablation were observed in the SEM images [21]. The blind-hole machining results of the oscillating machining method and conventional machining method are visually compared in Figure 15. Based on simulation research, it can be known that the protrusion at the bottom of the hole is formed by debris forming a low-speed zone, which is continuously sintered to form this structure. However, during oscillating machining, the low-speed zone problem is effectively solved, so the bottom of the hole will not form a protrusion.

### 3.6. Electrode Wear Analysis During Oscillating Machining

In conventional machining processes, as the machining depth increases, secondary discharge phenomena intensify due to deteriorating debris evacuation, causing significant radial dimensional changes in the electrode and tapered hole profiles. In contrast, the oscillation-assisted method reduces secondary discharge effects on the tool electrode, resulting in negligible radial wear. Consequently, this method eliminates machining dimensional errors caused by electrode radial deformation when processing workpieces of varying thicknesses.

## 4. Conclusions

To uncover the formation causes of machining defects in C_f_-ZrB_2_-SiC blind holes during micro-EDM and analyze the actual impact of the electrode-assisted oscillating device on the machining gap, a systematic analysis of machining flow field characteristics with and without the electrode-assisted oscillating device was conducted by comparing flow field dynamics and debris distribution. Specifically, CFD analysis clarified the underlying principles and yielded the following key findings:

(1) At a spindle speed of 3000 rpm and oscillation frequency of 60 rpm, the maximum flow velocity in the side gap under oscillation-assisted conditions reached 62 mm/s, surpassing the 47 mm/s achieved in conventional processing. The enlarged debris accommodation space facilitated efficient debris removal and minimized secondary discharges.
(2) Oscillation of the electrode dynamically shifted low-velocity zones in the bottom flow field, preventing localized debris buildup. Notably, while conventional methods exhibited near-stagnant flow velocities (approaching 0 mm/s) in minimum velocity regions, oscillation-assisted processing maintained a minimum velocity of 7.5 mm/s, ensuring continuous debris displacement.
(3) DPM simulations demonstrated that vortices in the oscillating flow field exerted a traction effect on debris particles. Residual debris on the bottom surface was substantially reduced, with only minor transient aggregations observed in localized areas. These accumulations dispersed as the electrode position changed, effectively mitigating long-term particle clustering.
(4) The oscillation-assisted device imparted higher velocities to electro-erosion products on the bottom surface, enabling rapid removal of newly generated debris from critical discharge zones. In contrast, conventional methods exhibited severe debris stagnation in edge and central regions, directly contributing to electrode edge wear, central depression, and geometric defects such as rounded corners and bottom surface protrusions.
(5) The oscillating machining method significantly reduced the secondary discharge effect on the tool electrode, resulting in extremely minor radial wear. Consequently, when using the oscillation machining method for continuous machining, it can ensure smaller machining dimensional errors.

### Further Research

In this study, the feasibility and advantages of the oscillating machining method have been further elucidated through fluid simulation. However, it is still worthwhile to conduct in-depth research from multiple aspects to further validate the universality of this method.

Firstly, this method has demonstrated its applicability in machining C_f_-ZrB_2_-SiC material. Given that EDM involves minimal force and is not restricted by material hardness, in theory, this method can also be applied to the machining of other metal or ceramic materials, but its specific performance remains to be verified through practical experiments.

In addition, when machining with the current-sized tool electrode, no significant flexural deformation was observed by in situ observation. So, this issue was not taken into account during the simulation process. However, if this method is applied to the machining of smaller-sized holes, smaller-diameter tool electrodes will be required. Whether the new electrodes can operate effectively under the control of the guides, whether they will fracture, or whether they will experience flexural deformation under the action of centrifugal force still necessitates further research.

Finally, this oscillating machining method also merits further optimization and expansion from a process perspective. Regarding the high-precision machining of irregular hole shapes, it is essential to investigate the feasibility of this method. By integrating simulation and experimental approaches, we can enhance the method’s capability in processing complex structures. Additionally, combining oscillating EDM with other auxiliary machining techniques allows us to explore the synergistic effects among different technologies and formulate appropriate integration strategies to maximize machining performance.

## Data Availability

The original contributions presented in this study are included in the article. Further inquiries can be directed to the corresponding authors.
